# The Role of Biologically Active Materials in Peri-Implant Diseases

**DOI:** 10.3390/jcm15134868

**Published:** 2026-06-23

**Authors:** Faustino Mercado, Carolina Loch

**Affiliations:** 1 Faculty of Dentistry, University of Otago, Dunedin 9016, New Zealand; carolina.loch@otago.ac.nz; 2 Sir John Walsh Research Institute, University of Otago, Dunedin 9016, New Zealand

**Keywords:** peri-implantitis, biologically active materials, growth factors, regeneration, evidence-based treatment

## Abstract

**Background/Objectives:** Peri-implant diseases, encompassing peri-implant mucositis and peri-implantitis, affect 43% and 18.8–23% of implant-bearing patients, respectively, representing significant clinical challenges in implant dentistry. While mechanical debridement remains foundational, biologically active materials offer promising adjunctive regenerative strategies. This narrative review synthesises current evidence regarding five biologically active materials: enamel matrix derivative (EMD), platelet-rich fibrin (PRF), fibroblast growth factor-2 (FGF-2), recombinant human platelet-derived growth factor-BB (rhPDGF-BB/GEM 21S^®^), and polynucleotide–hyaluronic acid combinations (Regenfast^®^). **Methods:** The relevant literature was identified using electronic databases, including MEDLINE, PubMed, Scopus, and Google Scholar. This review focused on clinical studies and randomised controlled trials with a minimum follow-up of six months investigating biologically active materials in peri-implant disease management. Material mechanisms, clinical efficacy, therapeutic limitations, and evidence quality were systematically evaluated. Attention was directed toward identifying genuine biological distinctions between peri-implant and periodontal disease contexts. **Results:** EMD demonstrates efficacy exclusively within multimodal surgical protocols, with isolated application yielding limited benefits. rhPDGF-BB shows superior periodontal regenerative capacity; however, dedicated peri-implantitis trials remain absent. FGF-2 exhibits paradoxical osteogenic suppression despite bone fill achievement, limiting peri-implant applicability. PRF and Regenfast^®^ demonstrate a mechanistically sound rationale yet lack substantive peri-implant disease validation. The critical findings revealed that peri-implant regeneration fundamentally differs from periodontal regeneration: implants lack periodontal ligament anatomy, rendering ligamentogenic differentiation-promoting agents biologically inappropriate. **Conclusions:** Contemporary biologically active materials demonstrate compelling periodontal efficacy yet remain inadequately validated for peri-implantitis management. This disparity reflects authentic biological distinctions rather than insufficient investigation. Until multicentre randomised controlled trials stratify efficacy across distinct peri-implant disease presentations, practitioners must prioritise evidence-based surgical fundamentals—meticulous decontamination, strategic grafting, and optimised wound healing—integrating biologically active materials judiciously within comprehensive, anatomy-respecting treatment protocols.

## 1. Introduction

Peri-implant diseases, encompassing both peri-implant mucositis and peri-implantitis, represent significant clinical challenges in implant dentistry [1]. These conditions range from reversible soft-tissue inflammation to irreversible bone loss surrounding osseointegrated implants, potentially compromising implant longevity and patient outcomes [2,3]. While mechanical debridement and surgical access remain foundational treatment approaches, the integration of biologically active materials has emerged as a promising adjunctive strategy to enhance tissue regeneration and arrest disease progression [4,5,6].

This narrative review synthesises current evidence regarding five biologically active materials utilised in peri-implant disease management: enamel matrix derivative (Emdogain^®^; EMD), platelet-rich fibrin (PRF), fibroblast growth factor-2 (FGF-2), recombinant human platelet-derived growth factor-BB (rhPDGF-BB)/GEM 21S^®^), and polynucleotide–hyaluronic acid combinations (Regenfast^®^). Each material represents a distinct biological approach, from naturally derived protein complexes to biotechnologically synthesised growth factors and regenerative platforms [4]. This review examines their origins, mechanisms of action, clinical efficacy in peri-implant applications, and potential contraindications, aiming to provide clinicians with evidence-based guidance for material selection and application in complex peri-implant defects.

Dental implants have revolutionised the management of edentulism, yet their widespread success is increasingly shadowed by biological complications that demand innovative therapeutic strategies [6]. Peri-implant mucositis, characterised by reversible soft-tissue inflammation, and peri-implantitis, defined by progressive, irreversible inflammatory bone loss, are an increasing global burden with substantial epidemiological impact [2,6]. The current evidence indicates that peri-implant mucositis affects approximately 43% of implant-bearing patients, while peri-implantitis prevalence ranges from 18.8% to 23% depending on assessment criteria, with epidemiological reviews suggesting that peri-implantitis affects approximately one in five to one in four implant patients overall, rendering these conditions among the most common biological complications in implant dentistry [6,7,8,9]. As implant placement accelerates worldwide, this parallel rise in biological complications has generated an urgent, unmet clinical demand for effective, predictable therapies capable of addressing the unique pathobiological characteristics of peri-implant diseases [10,11].

Managing peri-implantitis is fundamentally more challenging than treating periodontitis due to stark anatomical and biological disparities: implants lack a periodontal ligament and possess diminished vascular supply, while complex implant macro-designs, specific thread geometries, and micro-roughened surfaces severely complicate mechanical decontamination and regeneration [12,13,14,15]. Consequently, non-surgical therapy—though targeting essential biofilm disruption, mucosal inflammation reduction, and patient compliance improvement—remains insufficient for advanced disease, with clinical outcomes being notoriously modest due to restricted blind debridement access and the exceedingly rare achievement of true osseous refill; current evidence and consensus documents consistently conclude that non-surgical therapy yields highly limited long-term disease resolution [12,13,14]. Surgical intervention becomes necessary for deep, non-contained defects, providing critical visual access for thorough surface decontamination and enabling resective or reconstructive defect management [16,17]. Regenerative or reparative outcomes remain highly unpredictable due to immense clinical heterogeneity across implant surface characteristics, defect configurations, decontamination methods, biomaterial selection (grafting, membranes, and biologically active agents), and individual patient risk profiles. Current systematic reviews confirm that no single, universal surgical protocol has demonstrated clear, predictable superiority across all clinical scenarios for peri-implantitis [18,19,20,21].

This evidence gap has prompted substantial investigation into biologically active materials, including EMD, PRF, GEM 21S^®^, and Regenfast^®^, to facilitate bone and soft-tissue regeneration or repair within the compromised peri-implant environment [22,23,24,25]. These biologically active materials represent a promising frontier in peri-implantitis management, yet their comparative efficacy, cost-effectiveness, optimal application contexts, and role within comprehensive treatment algorithms remain poorly established, necessitating critical examination of current evidence, identification of agreements and disagreements among studies, clarification of material-specific limitations, and articulation of priority investigations required to translate biological plausibility into predictable clinical outcomes and evidence-based therapeutic recommendations.

## 2. Methods

A critical narrative synthesis methodology was implemented to comprehensively appraise evidence addressing therapeutic applications of biologically active materials within peri-implant disease contexts. The multiphase database interrogation included MEDLINE, PubMed, Scopus, and Google Scholar repositories, subsequently augmented via Cochrane Oral Health Group and Cochrane Library supplementary searches, ensuring exhaustive capture of original investigations and synthesised evidence assemblies. Standardised, predefined terminology was operationalised individually and via combinatorial permutations, maximising retrieval probability across heterogeneous nomenclatural conventions. The search terminology comprised semantic categories: disease nomenclature (peri-implantitis and peri-implant mucositis), material designations (enamel matrix derivative/EMD/Emdogain^®^, platelet-rich fibrin/PRF, fibroblast growth factor-2/FGF-2, recombinant human platelet-derived growth factor-BB/GEM 21S^®^, and Regenfast^®^), and chemical identifiers (polynucleotides and hyaluronic acid compounds).

### Eligibility Criteria and Screening Methodology

Inclusion Parameters: Human clinical investigations evaluating biologically active material efficacy within peri-implant disease management, prioritising randomised controlled trial designs with minimum six-month longitudinal observation intervals and English-language publication. Prospective case–control, comparative effectiveness, and cohort examinations were considered where randomised controlled trial evidence remained limited.

Exclusion Parameters: Laboratory-based investigations lacking clinical translatability (except animal histological studies substantiating clinical interpretation), studies with insufficient outcome specification (probing depth, attachment level, radiographic assessment, and adverse events), duplicate publications, and reports demonstrating inadequate methodological transparency. The sequential two-stage screening incorporated a preliminary title–abstract assessment identifying thematic relevance, followed by a comprehensive full-text evaluation against complete eligibility criteria. Reference list examination of prominent systematic reviews identified additionally overlooked publications. Standardised abstraction instruments captured study design, population characteristics, defect specifications, intervention parameters, temporal documentation, clinical outcomes (probing depth reduction, attachment level gain, radiographic bone changes, and bleeding suppression), secondary parameters (adverse events and quality-of-life metrics), and tissue-level endpoints. Evidence synthesis employed narrative analytical approaches with hierarchical weighting: randomised controlled trials received primary emphasis, followed sequentially by prospective cohort investigations, observational studies, and selective animal model inclusion where mechanistic evidence substantiated clinical frameworks.

## 3. Peri-Implantitis: A Biologically Distinct Lesion

### 3.1. Anatomical and Biological Distinctions Between Peri-Implantitis and Periodontitis

#### 3.1.1. Structural Architecture of Tooth–Tissue and Implant–Tissue Interfaces

Despite occupying a shared anatomical environment within the alveolar bone and oral mucosa, natural teeth and dental implants exhibit fundamentally divergent structural configurations and tissue interactions [11,15]. Natural teeth are secured to the alveolar bone and gingiva through a specialised fibrous apparatus comprising the periodontal ligament (PDL) and supracrestal connective tissue fibres, which establish a direct fibrous linkage between the cementum and alveolar bone. This attachment system develops concurrently with root formation during tooth development. The coronal boundary is demarcated by a thin junctional epithelium at the gingival margin, which maintains continuity with both the sulcular and oral epithelium [11,13].

In contrast, the peri-implant soft and hard tissues do not develop through conventional physiological mechanisms but rather through a post-surgical wound-healing trajectory [12]. The initial tissue trauma induced by the osteotomy procedure preceding implant placement initiates a cascade of bone-remodelling events, including lateral bone resorption adjacent to the implant body. Subsequent remodelling of both the bone–implant interface and the peri-implant mucosa extend over several weeks, during which a junctional epithelium progressively forms and the intervening connective tissue gradually adapts to the implant surface [12,13].

#### 3.1.2. Histoarchitectural Differences in Connective Tissue Organisation and Vascularity

The organisation of the connective tissue interface of these two systems structurally differs. While natural teeth are characterised by substantial anchorage fibres perpendicular to the tooth surface, the connective tissue surrounding implants lacks this integrated fibrous anchoring system. The collagen fibres within the peri-implant mucosa are predominantly oriented parallel to the long axis of the implant body [12,13]. Furthermore, the vascular density within the supracrestal connective tissue of the peri-implant mucosa is reduced compared with the corresponding periodontal region surrounding natural teeth [12,13].

#### 3.1.3. Osseointegration as the Mechanistic Basis of Implant Stability

These fundamental structural disparities necessitate a reconceptualisation of peri-implantitis as biologically distinct from periodontitis, rather than as a simple clinical analogue. The most significant distinction is that implants, being non-living biomaterials, cannot function through the dynamic mechanosensitive interplay characteristic of the natural tooth-supporting apparatus, namely, the integrated cementum, periodontal ligament, and lamina dura. Implant stability is maintained via osseointegration, an interfacial phenomenon of biomechanical and biochemical compatibility between a non-biological substrate (typically titanium) and living calcified tissue (bone) [12,13,15].

Beyond the intrinsic biological differences, implant-related design variables significantly modulate the tissue microenvironment surrounding the implant. These design factors include implant morphology and classification, thread geometry and surface topography, implant–abutment connection design, abutment configuration, and the material composition and structural arrangement of the prosthetic suprastructure [15,16,17,18]. These design characteristics not only influence the biological conditions of the surrounding tissues but also substantially complicate the chemical and mechanical decontamination of implant surfaces during therapeutic interventions [15,16,17,18].

### 3.2. Distinctive Pathophysiological Features of Peri-Implantitis Lesions

#### 3.2.1. Architectural Failure and Epithelial Discontinuity

Peri-implantitis lesions demonstrate three distinctive pathological characteristics that fundamentally differentiate them from periodontitis. First, the epithelial lining may fail to maintain continuity along the entire depth of the pocket; consequently, the apical third of the lesion frequently remains denuded of epithelial coverage, exposing severely inflamed connective tissue that is in direct contact with a densely organised microbial biofilm colonising the implant surface [12,13]. This architectural failure has profound implications for lesion progression.

#### 3.2.2. Loss of Barrier Function and Direct Osteoclastic Activation

Second, the anatomical absence of a periodontal ligament fundamentally compromises the defensive capacity of peri-implant connective tissue. Natural teeth possess a PDL-mediated barrier that spatially separates inflammatory cell infiltrates from the crestal bone margin. In contrast, peri-implant tissues lack this protective function, allowing direct contact between inflammatory cell populations and the crestal bone surface, where osteoclasts are characteristically mobilised and positioned along the bone margin in active peri-implantitis sites [11,12].

#### 3.2.3. Quantitative and Qualitative Differences in Inflammatory Infiltrates

Third, peri-implantitis and periodontitis differ significantly in both the dimensional scale and cellular composition of their inflammatory infiltrates. Histopathological investigations in human subjects have consistently demonstrated that peri-implantitis lesions exceed the volumetric extent of periodontitis lesions by more than twofold, while simultaneously harbouring disproportionately elevated numbers and densities of plasma cells, neutrophils, and macrophages [11,12,13].

#### 3.2.4. Mechanistic Determinants of Accelerated Disease Progression

The distinct histopathological profile of peri-implantitis lesions is largely attributable to the structural deficiencies intrinsic to implants, specifically, the absence of cementum, a functional periodontal ligament, and supracrestal attachment fibres. The inability to confine the inflammatory lesion in a location distant from the crestal bone, combined with the failure to establish an intact epithelial barrier that would shield the inflamed connective tissue from biofilm-derived antigens and virulence factors, accounts for the non-linear and accelerating disease trajectory observed in peri-implantitis, as well as its substantially more rapid progression compared with periodontitis [12,15,21].

#### 3.2.5. Molecular and Microscopic Evidence of Enhanced Inflammatory Susceptibility

Significant microscopic and molecular distinctions have been identified between peri-implant tissues and intact periodontium. The reduced vascularisation and parallel collagen fibre orientation characterising peri-implant tissues render these tissues substantially more susceptible to inflammatory activation than their periodontal counterparts [26]. This heightened inflammatory susceptibility has been substantiated via immunohistochemical analysis, which has documented increased accumulation of inflammatory infiltrate, elevated expression of nitric oxide synthase, increased vascular endothelial growth factor (VEGF), augmented lymphocyte and leukocyte populations, and heightened Ki67 expression within peri-implantitis lesions [27].

Matrix metalloproteinase (MMP) concentrations, particularly the collagen-degrading enzyme MMP-8, are substantially elevated in peri-implant lesions relative to periodontal lesions, a finding with potential diagnostic utility [28,29]. At the immunological level, peri-implantitis tissues demonstrate an increased proportion of B lymphocytes and elevated concentrations of the pro-inflammatory cytokines interleukin-1β (IL-1β) and tumour necrosis factor-α (TNF-α), alongside elevated concentrations of the T-helper 2 (Th2) cytokine interleukin-4 (IL-4) and B-cell-activating factor (BAFF) [11]. This cytokine profile reflects the compromised capacity of peri-implant tissues to mount an effective immunological barrier compared with the natural tooth-supporting apparatus.

### 3.3. Distinct Microbial Ecology in Peri-Implant Pathology

#### 3.3.1. Comparative Microbiological Characteristics

The microbial communities associated with peri-implantitis and periodontitis exhibit fundamental compositional differences. In periodontitis, dysbiosis of the subgingival microbiome is the primary pathogenic driver of disease initiation and progression, characterised by selective overgrowth of obligate anaerobic pathogens such as *Porphyromonas gingivalis*, *Tannerella forsythia*, and *Aggregatibacter actinomycetemcomitans* [14]. In contrast, the subgingival biofilm in peri-implantitis is characterised by substantially greater density and microbial diversity, encompassing a markedly broader spectrum of pathogenic bacterial species [14].

#### 3.3.2. Dynamic Microbiome Shifts and Species Enrichment Patterns

Integrated microbiological and immunological investigations have elucidated distinct pathogenic mechanisms in peri-implantitis. Longitudinal 16S ribosomal RNA (rRNA) gene sequencing has demonstrated dynamic temporal shifts in the peri-implant microbiome correlating with disease progression and therapeutic recovery, with characteristic enrichment of species including *Streptococcus parasanguinis*, *Streptococcus mutans*, *Cutibacterium acnes*, and *Aerococcus viridans* [30]. Simultaneously, heightened transcriptional expression of genes encoding bacterial flagellar proteins, chemotactic machinery, and diverse metabolic pathways has been documented, indicating a shift toward a more metabolically active and motile bacterial community [30].

#### 3.3.3. Titanium Particle-Mediated Modulation of Microbial Structure and Immune Response

Evidence indicates that titanium particles, generated via implant wear and surface degradation, significantly modulate microbial community composition and are positively correlated with Th17 lymphocyte-polarised inflammatory dominance within peri-implant crevicular fluid [31]. This observation suggests a mechanistically distinct microenvironment in peri-implantitis compared with periodontitis, wherein a “microbial mosaic and inflammatory core” mechanism drives more aggressive tissue destruction [31]. This pathogenic mechanism necessitates concurrent intervention at two critical levels: disruption of the biofilm architectural matrix and modulation of local immune cascade activation for clinical therapeutic success [31].

#### 3.3.4. Distinct Inflammatory Phenotypes and Clinical Implications

Recent characterisations have delineated two distinct inflammatory patterns within peri-implantitis lesions: a chronic inflammatory response and a hypersensitivity-type inflammatory reaction. Both are potentially modulated by the presence of titanium particles, together accounting for the more aggressive tissue-destructive phenotype observed in peri-implantitis compared with periodontitis [31]. The clinical translation of these microbiological and immunological findings indicates the need for exceptionally thorough and efficacious decontamination of infected implant surfaces while simultaneously minimising the generation of titanium particles within the peri-implant healing microenvironment [31].

#### 3.3.5. Dysbiosis-Driven Loss of Homeostatic Host–Microbial Transactions

These findings further support the hypothesis that microbial dysbiosis within the peri-implant sulcus actively disrupts the homeostatic host–bacterial transactions that maintain tissue health, instead driving a pathological shift toward chronic programming of a non-healing wound phenotype [32]. This dysbiosis-driven transition to a non-healing state typically necessitates surgical intervention and intensive management protocols, distinguishing peri-implantitis from its periodontal counterpart in terms of therapeutic complexity and treatment demands [32].

## 4. Enamel Matrix Derivative (Emdogain; EMD)

### 4.1. Developmental Origins and Biological Foundation

The therapeutic potential of enamel matrix derivative was discovered via a systematic investigation demonstrating that proteins composing the enamel matrix retain an inherent capacity to recapitulate the developmental processes governing periodontal tissue formation [33]. This biological preparation functions as an organised macromolecular assembly that orchestrates endogenous cellular activities rather than providing inert structural scaffolding; it is derived via extraction from porcine tooth enamel and characterised predominantly by amelogenin constituents supplemented with additional enamel-associated protein fractions [34]. The underlying biological principle resides in EMD’s demonstrated ability to facilitate the generation of novel cementum, periodontal ligament structures, and alveolar bone through pathways involving both direct cellular interaction and indirect signalling mechanisms [33,35].

### 4.2. Cellular and Molecular Mechanisms of Therapeutic Action

The operational framework of EMD encompasses several coordinated biological processes working synergistically. In the first instance, amelogenin components augment the proliferative capacity and phenotypic maturation of osteoblasts while simultaneously exerting regulatory control over the osteoprotegerin/RANKL signalling axis, consequently suppressing the bone-resorptive activities of osteoclasts [36,37]. Secondarily, the preparation stimulates the migratory behaviour of fibroblasts and facilitates enhanced production of extracellular matrix components, thereby enabling restoration of periodontal ligament tissue architecture [38]. Additionally, EMD demonstrates antimicrobial properties effective against pathogenic periodontal microorganisms, accomplishing a favourable compositional shift in microbial communities toward Gram-positive aerobic bacterial species that exert reduced destructive potential on implant-surrounding tissues [39,40]. Furthermore, the protein assembly facilitates cellular adhesion phenomena and promotes phenotypic specification of periodontal ligament-derived cells, thereby establishing an environment conducive to restorative tissue development [34,41].

### 4.3. Clinical Applications and Efficacy in Peri-Implant Pathology

EMD’s application in the therapeutic management of peri-implant disease demonstrates heterogeneity in clinical outcomes and remains substantially constrained compared with its established effectiveness in managing primary periodontal pathology [4]. The historical extrapolation of EMD utilisation from demonstrated periodontal success encountered fundamental biological obstacles. Specifically, the structural absence of authentic periodontal ligament attachment in the implant–tissue interface restricts EMD’s regenerative capacity within peri-implant contexts [42]. Accumulating evidence indicates that EMD achieves meaningful therapeutic benefit exclusively within the framework of comprehensive, multi-component regenerative surgical protocols [3,5].

The investigation series documented by Froum and colleagues (2015) demonstrates that multimodal regenerative protocols—incorporating EMD alongside platelet-derived growth factor, osseous replacement substances, and occlusive barrier technologies—produce sustained clinical improvements in peri-implantitis: probing depth reductions spanning 5.1–5.4 mm and concurrent marginal bone-level improvements of 3.0–3.75 mm. Implant stability was preserved at all 51 sites affected by peri-implantitis, and these improvements were maintained throughout extended observation periods of 3–7.5 years [3]. In contrast, an investigation conducted by Isehed and associates (2016) employing a randomised controlled methodology examined EMD in isolation and documented that bone regeneration manifested in radiographic assessment via multivariate statistical analysis (enhanced marginal bone dimension) occurred without corresponding amelioration of established clinical disease indicators, including probing depth improvement, bleeding suppression, or microbial control according to univariate analytical approaches [42]. This distinction—the discordance between radiographic bone reconstitution and persistent inflammatory manifestations—demonstrates EMD’s incomplete therapeutic scope when deployed as a singular adjunctive intervention in peri-implantitis and periodontitis [3,5,43].

Prospective longitudinal analysis conducted by Mercado and collaborators (2018) integrating EMD with demineralised bovine bone matrix, collagen-based biomaterials, and antimicrobial doxycycline administration achieved statistically robust reductions in probing measurements and bone loss quantification; nevertheless, clinical therapeutic success was restricted to 56.6% of the treated population [5]. A comprehensive systematic evaluation and quantitative synthesis performed by Donos with colleagues (2023), synthesising evidence from seven randomised controlled investigations, concluded that while regenerative reconstruction methodologies exceed the therapeutic yield of traditional flap access techniques, no individual reconstructive methodology, including strategies incorporating EMD, demonstrated superiority across the therapeutic spectrum. Current evidence has proven to be insufficient to designate EMD as a mandatory or distinctly superior component within peri-implantitis treatment frameworks [4].

### 4.4. Consolidated Evidence and Three Core Principles

Principle 1. Obligate multimodal integration: The clinical benefit accruing from EMD application in peri-implantitis treatment fundamentally relies on a comprehensive surgical protocol architecture [3]. Integration into therapeutic protocols encompassing microbiological decontamination of implant surfaces, supplemental growth factor administration (emphasising platelet-derived growth factor), osseous augmentation materials, and protective membrane systems enables EMD to contribute meaningfully to sustained bone reconstitution [3]; conversely, when administered in isolation, the magnitude of therapeutic benefit diminishes substantially [44].

Principle 2. Tissue-selective therapeutic profile: EMD demonstrates preferential enhancement of radiographic disease parameters (marginal bone level restoration) while demonstrating inconsistent resolution of inflammatory disease manifestations, including probing depth, gingival haemorrhage, and purulent exudation [5,44]. This compartmentalisation of effects, selective osseous benefit coupled with incomplete inflammatory suppression, suggests that EMD selectively addresses bony architectural defects while potentially leaving certain soft-tissue inflammatory components unresolved [5,44].

Principle 3. Absence of disease-modifying monotherapy evidence: Existing clinical evidence fails to substantiate EMD’s utility as a standalone disease-modifying pharmacological agent [4]. Conversely, evidence supports consideration of EMD as a potentially valuable therapeutic component integrated within broader regenerative treatment paradigms, with clinical deployment determined by anatomical defect characterisation, individualised patient variables, and overarching surgical design philosophy rather than as a universally applicable mandate [4].

## 5. Platelet-Rich Fibrin (PRF) in Implant Dentistry

### 5.1. Foundational Principles and Biological Rationale

Platelet-rich fibrin constitutes an autologous biomaterial-processing technology wherein venous blood undergoes centrifugal separation, generating a three-dimensional fibrin scaffold matrix populated with aggregated platelets, incorporated leukocytic elements, and entrapped soluble growth factor mediators [45]. Distinguished from preceding platelet concentration methodologies, PRF’s formulation excludes supplemental anticoagulant chemistry and exogenous thrombin administration, thereby maintaining entirely autologous biological composition while enabling sustained temporal release of bioactive growth factors throughout extended healing intervals [46]. The self-derived biological nature of PRF, combined with its physiologically determined release kinetics, fundamentally distinguishes it from synthetically manufactured recombinant growth factor preparations administered via external introduction [47].

Contemporary systematic evaluations have documented that PRF’s therapeutic utility extends across diverse oral surgical applications, encompassing alveolar bone regeneration, periodontal defect management, and dental implant site development [22]. The biological rationale underlying PRF implementation rests upon its capacity to deliver multiple bioactive mediators within a physiologically compatible matrix, thereby recreating fundamental aspects of the natural wound-healing cascade without exogenous pharmaceutical supplementation. This autologous approach addresses significant limitations inherent to recombinant growth factor preparations, including cost considerations, immunological variability, and regulatory complexity, whilst maintaining mechanistic efficacy grounded in established haemostatic and regenerative biology [22,45]. Within implant dentistry specifically, PRF has gained clinical recognition for its dual capacity to simultaneously augment osseous regeneration and optimise peri-implant soft-tissue architecture, positioning it as a versatile adjunctive biomaterial in comprehensive implant treatment planning [25].

### 5.2. Biological Mechanisms and Operational Pathways

PRF accomplishes its therapeutic effects via multiple coordinated biological mechanisms functioning in parallel. The fundamental scaffold function originates from the fibrin matrix architecture, which supplies three-dimensional geometric organisation facilitating directional cellular migration and coordinated tissue reconstruction, simultaneously serving as a physical support structure and a regulated-delivery mechanism for bioactive substances [48]. The sequestered platelet populations and incorporated leukocytic cells undergo gradual release of diverse growth factor species, including platelet-derived growth factor (PDGF), vascular endothelial growth factor (VEGF), fibroblast growth factor (FGF), transforming growth factor-beta (TGF-β), and insulin-like growth factor-1 (IGF-1), throughout an extended 7–14-day window [49,50]. This protracted release mechanism generates a persistent paracrine signalling microenvironment, contrasting sharply with the rapid biological clearance characteristic of administered exogenous growth factor preparations [51].

Additionally, PRF exerts immunoregulatory effects mediated primarily through the integrated leukocytic component within the fibrin lattice, promoting resolution-phase inflammatory responses and directing macrophage phenotypic polarisation toward tissue-reconstruction-promoting cellular states [52]. The sequential liberation of VEGF and FGF promotes neovascular formation, establishing functional microvascular networks essential for physiological tissue repair processes and successful osseointegration within regenerative defect regions [53]. Recent mechanistic investigations have further elucidated that PRF’s fibrin scaffold undergoes progressive remodelling throughout the healing phase, creating dynamic microenvironmental conditions that optimise sequential cellular recruitment and phenotypic differentiation [22]. The leukocytic-enriched composition contributes substantially to immunomodulatory functions, distinguishing PRF from earlier-generation platelet-concentrate preparations; this immunological dimension may enhance tissue healing efficiency in clinically challenging scenarios characterised by compromised microvascular perfusion or chronic inflammatory states [22]. Notably, PRF-derived fibrin has demonstrated capacity to stimulate collagen synthesis in both periodontal ligament and osteoblastic cells in vitro, supporting its clinical utility in tissue regeneration contexts [41].

### 5.3. Methodological Heterogeneity and Clinical Evidence in Implant Applications

Substantial heterogeneity exists within contemporary PRF preparation methodologies, significantly impacting reproducibility and comparative analysis of clinical investigations [22]. Centrifugal protocols vary substantially regarding gravitational force (relative centrifugal force values ranging from 400 to 2700 g), temporal duration, temperature parameters, and tube composition, generating mechanistic inconsistencies in final fibrin architecture, cellular composition, and growth factor concentrations [22]. This methodological divergence fundamentally compromises evidence synthesis across published investigations, as distinct PRF formulations may demonstrate substantially different biological potencies and regenerative capacities [22]. Comparative analyses of growth factor release patterns from conventional PRF and advanced-PRF (A-PRF) formulations have documented significant quantitative and temporal variations, underscoring the critical importance of protocol standardisation for clinical application [54].

Systematic reviews examining PRF’s clinical efficacy specifically within implant dentistry contexts have documented moderate-to-substantial therapeutic benefit. Investigations evaluating PRF in alveolar bone preservation following tooth extraction demonstrate consistent benefits regarding dimensional stability and bone volume maintenance, supporting routine implementation in extraction socket management protocols [22]. Dental implant site development represents an increasingly documented application, wherein PRF is utilised either as a standalone regenerative approach or in combination with particulate bone grafting materials to optimise bone volume in anatomically compromised implant sites [22]. Clinical investigations indicate that PRF-augmented bone regeneration procedures generate comparable or superior osseous defect fill relative to conventional grafting methodologies alone, with particular utility in posterior maxillary regions demonstrating restricted alveolar bone dimensions [22]. Furthermore, PRF application to implant surfaces prior to insertion and utilisation as a soft-tissue augmentation material have demonstrated beneficial effects on early osseointegration, peri-implant soft-tissue architecture, and post-operative inflammatory responses [22].

### 5.4. Clinical Implementation and Evidence in Peri-Implant Disease Management

The therapeutic application of PRF within peri-implant pathology management represents an emerging clinical application with expanding but incompletely characterised evidence foundation. Recent randomised controlled trials have established efficacy of leukocyte- and platelet-rich fibrin in augmenting peri-implant mucosal thickness in edentulous patients treated with mandibular implant-retained overdentures, demonstrating clinically meaningful soft-tissue dimensional gains [55]. Additionally, prospective investigations evaluating subgingival irrigation with injectable PRF (i-PRF) following non-surgical peri-implantitis treatment have documented promising therapeutic outcomes, suggesting potential utility in disease resolution protocols [56].

Notwithstanding these recent developments, comprehensive prospective randomised controlled investigation protocols specifically evaluating PRF’s therapeutic efficacy in peri-implant mucosal mucositis or advancing peri-implantitis conditions remain limited [57]. Foundational laboratory investigations demonstrate PRF’s biological capacity to stimulate bone neogenesis within implant-adjacent tissues and facilitate soft-tissue wound-healing processes; nevertheless, these experimental studies do not provide definitive clinical evidence regarding disease resolution in scenarios involving established peri-implant inflammatory disease coupled with progressive bone resorption [48]. Systematic reviews specifically addressing PRF’s efficacy in peri-implant soft-tissue outcomes indicate insufficient evidence quantity for definitive recommendations, although preliminary investigations suggest potential beneficial effects on soft-tissue dimensional stability and healing kinetics [57]. The biomaterial has gained recognition for utility predominantly in augmentative procedures addressing peri-implant soft-tissue thickness and osteoconductive applications facilitating bone regeneration in implant-adjacent anatomical sites; however, prospective randomised investigation protocols specifically targeting the therapeutic management of active peri-implant disease processes represent an incomplete evidence domain [22,25].

### 5.5. Consolidated Understanding and Clinical Implications

Principle 1. Inherent Biocompatibility Through Autologous Origin: The autologous derivation of PRF fundamentally eliminates immunological reactivity complications accompanying exogenously administered synthetic recombinant protein preparations, affording significantly improved tissue compatibility and enhanced economic efficiency within contemporary implant dentistry practice environments [48]. This biological advantage is particularly relevant in immunologically compromised patient populations or scenarios requiring repeated therapeutic application in complex implant cases [22].

Principle 2. Temporally Extended Bioactive Compound Availability with Standardisation Requirements: The fibrin matrix substrate permits gradual liberation of sequestered growth factor mediators throughout a 1–2-week therapeutic window, creating a distinctive temporal delivery profile that diverges from rapid systemic clearance observed with directly administered soluble exogenous growth factor formulations [53]. However, substantial heterogeneity in PRF preparation protocols significantly influences final biological potency and regenerative capacity; standardisation of centrifugal parameters, temporal duration, and tube composition remains essential for improved reproducibility and clinical reliability within implant surgical practice [22]. This protracted availability mechanism may enhance cellular response optimisation, particularly within anatomically challenging defect configurations demonstrating restricted perfusion capacity [22,48].

Principle 3. Emerging Evidence Base for Peri-Implant Applications with Continued Investigation Requirements: Recent randomised controlled trials demonstrate PRF efficacy in augmenting peri-implant soft-tissue dimensions and facilitating non-surgical disease treatment outcomes; however, the evidence foundation remains incomplete regarding comprehensive disease resolution in advanced peri-implantitis scenarios [55,56]. The theoretical mechanistic advantages supporting PRF application in peri-implant therapy are increasingly supported by empirical verification through prospective investigations, yet substantial research gaps persist [57]. Contemporaneous clinical utilisation patterns have evolved beyond pure extrapolation from periodontal literature, incorporating implant-specific evidence; however, standardised clinical protocols and outcome measurement methodologies require further development [45,56].

PRF represents a mechanistically sound and biologically efficacious therapeutic approach with substantive documentation supporting its utility in implant site development, alveolar bone preservation, and emerging evidence for peri-implant soft-tissue augmentation and disease management [22,55,56]. Its clinical implementation in implant dentistry is supported by favourable safety profiles, economic efficiency, and demonstrated biological efficacy across diverse applications. However, methodological standardisation, systematic evidence synthesis, and prospective investigation protocols specifically targeting complex peri-implant disease scenarios remain essential prerequisites for establishing definitive evidence-based clinical recommendations. Future investigations must prioritise protocol standardisation and comparative effectiveness analysis to fully characterise PRF’s role within contemporary implant therapeutic paradigms [22,56,57].

## 6. Fibroblast Growth Factor-2 (FGF-2)

### 6.1. Developmental Evolution and Biological Characterisation

FGF-2, designated within scientific nomenclature as basic-FGF, functions as a critical signalling molecule orchestrating cellular proliferation, directional migration, and phenotypic differentiation processes within mesenchymal tissue compartments [53]. FGF-2 attained significant clinical recognition following Japan’s regulatory authorisation in 2016 of a lyophilised basic fibroblast growth factor preparation (Trafermin) designated for therapeutic application in periodontal tissue reconstruction [58,59]. Experimental investigations conducted within nonhuman primate and canine biological models established fibroblast growth factor-2’s capacity to accomplish comprehensive periodontal tissue regeneration encompassing newly synthesised cementum deposition, functionally aligned periodontal ligament fibre organisation, and alveolar bone neogenesis [58,60].

Investigative discoveries have documented concentration-dependent effects of fibroblast growth factor-2 on osteogenic phenotypic maturation within periodontal ligament-derived stem cell populations; in vitro studies indicate inhibitory effects on osteogenic differentiation markers despite concurrent volumetric bone accumulation observed in defect site healing [61,62]. These observations suggest multifactorial regenerative mechanisms that extend beyond direct osteoblast stimulation and warrant further mechanistic investigation. This apparent dichotomy has prompted development of compensatory modification approaches combining fibroblast growth factor-2 with biomaterial carriers. Contemporary research has evaluated basic fibroblast growth factor formulations with sulfonated chitosan oligosaccharide, a polymeric scaffold that may preserve fibroblast growth factor-2 bioactivity, maintain cell proliferative capacity, and support angiogenic and ligament-forming activities [61,62]. However, the clinical significance of these in vitro observations in actual bone regeneration remains incompletely characterised and requires validation through prospective clinical investigation.

### 6.2. Cellular and Molecular Mechanisms of Therapeutic Activity

Fibroblast growth factor-2 demonstrates documented angiogenic capacity and mitogenic effects on mesenchymal progenitor cell populations, promoting enhanced fibroblast proliferative activity, amplified extracellular matrix component production including hyaluronic acid and osteopontin deposition, and coordinated vascular neogenesis [24]. In the primary mechanism, fibroblast growth factor-2 binds to and activates fibroblast growth factor receptor family members expressed on periodontal ligament cell surfaces, thereby initiating intracellular signal transduction cascades that promote proliferative activity and matrix component synthesis [62,63]. In the secondary mechanism, fibroblast growth factor-2 stimulates neovascularisation via endothelial cellular activation and upregulation of vascular endothelial growth factor expression, establishing functional microvascular networks essential for defect site healing and tissue integration [24,64]. The tertiary mechanism involves fibroblast growth factor-2’s documented promotion of ligament-specific differentiation programs; preclinical observations suggest enhanced periodontal ligament tissue regeneration relative to isolated osseous regeneration outcomes, though the underlying biological mechanisms require further elucidation [58,64]. The quaternary mechanism encompasses demonstrated safety parameters, including absence of documented anti-fibroblast growth factor-2 antibody formation or identifiable systemic toxicological manifestations across clinical trials, thereby supporting therapeutic deployment [58].

### 6.3. Contemporary Clinical Evidence and Therapeutic Applications in Peri-Implant Disease

FGF-2’s clinical applicability within peri-implant pathology management remains limited by available clinical evidence. Phase III prospective clinical investigations demonstrated FGF-2’s effectiveness in periodontal osseous defects, achieving volumetric bone regeneration at 50.6% of original defect dimensions at 36-week observation intervals compared with 21.6% in placebo-treated groups [58,65]. Comparative effectiveness analyses indicated FGF-2 generated 1.93 mm of linear alveolar bone gain compared with 1.36 mm with enamel matrix derivative preparations [58]. However, clinical attachment level improvements demonstrated restricted magnitude (~2 mm) across treatment groups, mirroring patterns documented with alternative growth factors and suggesting enhanced osseous regeneration without proportional soft-tissue component regeneration [58].

Critically, dedicated prospective clinical investigations specifically evaluating FGF-2’s effectiveness in peri-implant mucosal inflammation or established peri-implantitis remain absent from the peer-reviewed literature [4]. While FGF-2’s periodontal regenerative capacity suggests potential applicability in peri-implant contexts, current clinical implementation relies on extrapolated evidence rather than direct empirical validation. The documented concentration-dependent effects on osteogenic differentiation may influence clinical outcomes in bone-deficient peri-implant sites. Any future clinical applications in peri-implant disease would necessitate independent prospective investigation before responsible clinical integration [61].

### 6.4. Consolidated Understanding and Three Fundamental Principles

Principle 1. Concentration-Dependent Osteogenic Effects: FGF-2 exhibits concentration-dependent effects on bone morphogenetic protein-mediated osteogenic differentiation pathways in vitro; however, the clinical significance of these observations and mechanisms underlying bone volume accumulation at defect sites require further investigation [61].

Principle 2. Ligament-Specific Differentiation Activity: FGF-2 demonstrates in vitro promotion of ligament-specific phenotypic differentiation programs, potentially accounting for superior periodontal ligament regeneration outcomes in natural dentition contexts [58]. The applicability of this tissue-selective activity within peri-implant therapeutic paradigms, where periodontal ligament formation is biologically unachievable, remains speculative and unsupported by clinical evidence [58].

Principle 3. Absence of Clinical Validation in Peri-Implant Disease: The peer-reviewed literature contains no prospective clinical investigations examining fibroblast growth factor-2’s effectiveness in peri-implant mucosal inflammation or peri-implantitis [4]. Current extrapolation from periodontal applications represents a theoretical hypothesis requiring independent clinical validation before responsible implementation.

FGF-2 demonstrates documented therapeutic effectiveness in periodontal regeneration applications; however, specific peri-implant disease applications lack direct clinical evidence and remain within investigational parameters. The concentration-dependent effects on cellular differentiation pathways warrant cautious interpretation pending further mechanistic clarification and prospective clinical investigation [61,66].

## 7. Recombinant Human Platelet-Derived Growth Factor-BB (rhPDGF-BB) and GEM 21S^®^

### 7.1. Historical Evolution and Biotechnological Development

rhPDGF-BB comprises a biotechnologically manufactured isoform of platelet-derived growth factor-BB that replicates the structural characteristics of endogenously occurring molecules while demonstrating enhanced biological activity via amplified osteoblast receptor affinity mechanisms [67,68]. The initial scientific recognition of platelet-derived growth factor’s capacity to facilitate periodontal tissue regeneration emerged from investigations conducted by Lynch and colleagues, who documented enhanced periodontal regeneration outcomes achieved via strategic combinations of platelet-derived growth factor and insulin-like growth factor preparations [67]. Early clinical implementation approaches utilised autologous platelet concentrate preparations derived directly from patient blood; however, the inherent limitations associated with standardisation variability and unpredictable immunogenic responses prompted investigative development of recombinant protein formulations capable of delivering consistent dosage administration while substantially reducing immunogenic complication risks [69].

GEM 21S^®^ merits particular analytical attention as a specific clinical product formulation, notwithstanding its fundamental composition as an rhPDGF-BB derivative [70]. The product’s regulatory authorisation via the FDA and extensive clinical adoption patterns within periodontal regenerative applications contrast with its comparatively restricted clinical validation in peri-implant disease contexts [70,71]. The difference between these applications does not originate from divergent biological mechanisms; both derive therapeutic benefit from identical rhPDGF-BB compositions. Instead, these applications show differential accumulation of clinical evidence and distinct regulatory authorisation frameworks [4]. GEM 21S^®^ represents a solitary FDA-authorised growth factor-based regenerative therapeutic designated for periodontal osseous defects; however, this regulatory authorisation status does not include therapeutic applications within peri-implant disease management contexts [4,70]. Healthcare practitioners implementing GEM 21S^®^ for peri-implant therapeutic applications engage in extra-label clinical practice lacking adequate evidence-based support [4]. Clinicians must acknowledge that off-label utilisation of GEM 21S^®^ in peri-implantitis management constitutes deviation from FDA-approved indications, thereby establishing medicolegal liability exposure, increased professional liability risk, and potential informed consent complications requiring explicit patient disclosure regarding therapeutic evidence limitations, unapproved status, investigational nature of the application, and absence of regulatory safety validation within this specific disease context [4].

### 7.2. Fundamental Biological Paradox: Delivery Vehicle Complications

A critical biological contradiction appeared during systematic product development investigations: β-TCP monotherapy paradoxically suppressed bone-healing processes, potentially attributable to excessive accumulation of calcium and phosphate ionic species exceeding the physiological tolerance thresholds of osteoblastic cellular populations [72]. Furthermore, controlled investigations examining growth factor liberation kinetics revealed substantial discordance between laboratory-determined release patterns and physiologically observed behaviour [72]. Laboratory-based in vitro investigations demonstrated rapid rhPDGF-BB liberation, with ninety per cent release accomplishment within ten-minute intervals and complete release by ninety minutes. In contrast, in vivo investigations utilising rat calvarial osseous models demonstrated substantially prolonged retention, with fifty per cent retention loss within thirty-minute intervals and only ten per cent persistence remaining at seventy-two-hour observation intervals [72]. This fundamental discrepancy suggests that β-TCP functions via physical entrapment mechanisms rather than chemical adsorption processes for rhPDGF-BB sequestration, potentially compromising the achievement of therapeutically prolonged growth factor delivery profiles [72].

### 7.3. Cellular and Molecular Mechanisms of Therapeutic Action

rhPDGF-BB accomplishes therapeutic effects via multiple distinct biological operational pathways. The initial mechanism involves direct mitogenic stimulation of osteoblast populations, promoting enhanced cellular proliferation and osteogenic phenotypic maturation by engaging high-affinity cell surface receptor-binding interactions [67,69]. The secondary mechanism encompasses indirect amplification of paracrine signalling via stimulation of macrophage-resident growth factor synthesis and secretion, thereby generating amplified therapeutic effects extending beyond direct osteoblastic cellular activation [73]. The tertiary mechanism involves PDGF’s potent chemotactic activity, recruiting mesenchymal stem cell and progenitor cell populations toward defect sites, thereby establishing cellular populations essential for accomplishing regenerative tissue development [67,69]. The quaternary mechanism encompasses PDGF’s distinctive capacity to simultaneously promote osteogenic differentiation pathways and soft-tissue proliferative responses, thereby distinguishing this growth factor from alternative preparations demonstrating predominantly ligamentogenic or skeletal tissue-specific bias [73].

### 7.4. Contemporary Evidence and Clinical Applications in Peri-Implant Disease

GEM 21S^®^ demonstrates substantial clinical effectiveness within periodontal intrabony osseous defect management [70]. Multicentric prospective randomised investigation protocols established the therapeutic effectiveness of GEM 21S^®^ formulations, documenting significantly superior clinical attachment level gains and volumetric bone fill achievement compared with placebo-treated controls and alternative biomaterial scaffolds [70]. At two-week observation intervals, rhPDGF-BB-containing defects demonstrated approximately double the mineralised bone formation magnitude compared with untreated controls and approximately ten-fold greater osseous formation relative to β-TCP monotherapy applications [70].

Conversely, peri-implant therapeutic applications represent a substantial clinical evidence deficiency. A preliminary investigation conducted within a sheep experimental model evaluated GEM 21S^®^ application in critical dimension circumferential peri-implant osseous defects, documenting superior bone regeneration outcomes compared with natural blood clot healing and β-TCP monotherapy approaches [74]. Nevertheless, prospective clinical investigation protocols specifically designed to evaluate rhPDGF-BB or GEM 21S^®^ therapeutic effectiveness within peri-implant mucosal inflammation or advanced peri-implantitis disease contexts remain absent from the peer-reviewed literature, substantially limiting the capacity to establish evidence-based clinical therapeutic recommendations for these specific disease conditions [4].

Recent investigative developments demonstrate nascent therapeutic potential within peri-implant soft-tissue augmentation applications [75]. A prospective randomised controlled investigation protocol directly comparing vascularised collagen biomaterial matrix supplemented with rhPDGF-BB against conventional connective tissue autograft approaches for managing peri-implant soft-tissue dehiscence deficiencies documented therapeutic effectiveness; nevertheless, extended longitudinal clinical outcome documentation and comparative effectiveness assessment relative to established surgical protocols necessitate continued investigation [75].

### 7.5. Consolidated Understanding and Three Fundamental Principles

Principle 1. Comprehensive Regenerative Capacity: rhPDGF-BB demonstrates superior clinical therapeutic effectiveness in accomplishing comprehensive periodontal tissue regeneration via distinctive dual-capacity mechanisms enabling synchronous achievement of osteogenic differentiation and soft-tissue proliferation responses [70,73]. This mechanistic profile differentiates rhPDGF-BB from alternative growth factor preparations exhibiting predominant bias toward ligamentogenic regeneration or isolated skeletal tissue development [70].

Principle 2. Biomaterial Vehicle Delivery Paradox: GEM 21S^®^ formulations employ β-TCP as the rhPDGF-BB biological delivery vehicle; conversely, β-TCP monotherapy paradoxically suppresses osseous healing processes [72]. This biological contradiction, utilising an osteoinhibitory biomaterial scaffold as the delivery carrier for an osteogenic growth factor, reflects complex delivery liberation kinetics and ionic concentration accumulation mechanisms that necessitate further mechanistic investigation for comprehensive biological understanding [72].

Principle 3. Insufficient Clinical Evidence for Peri-Implant Disease: Despite established clinical efficacy within periodontal regenerative contexts, dedicated prospective clinical investigation protocols specifically evaluating the therapeutic effectiveness of GEM 21S^®^ or rhPDGF-BB in peri-implant mucosal inflammation or advanced peri-implantitis disease remain conspicuously absent [4]. Peri-implant soft-tissue augmentation applications demonstrate emerging therapeutic promise; however, these applications require additional validation investigations [75].

rhPDGF-BB demonstrates superior clinical therapeutic efficacy within periodontal regenerative applications [70,73]; nevertheless, peri-implant disease management treatment applications remain insufficiently validated despite theoretical mechanistic promise [4].

## 8. Regenfast: Polynucleotides and Hyaluronic Acid Platform

### 8.1. Foundational Composition and Mechanistic Architecture

Regenfast^®^ constitutes an innovative regenerative therapeutic platform engineered by strategically combining two biochemically distinct biological components—polynucleotides and hyaluronic acid—which collectively generate synergistic tissue restoration mechanisms [76]. Polynucleotides, comprising long-chain deoxyribonucleic acid polymeric structures derived from fish spermatic tissues, engage and activate adenosine A_2_A cell surface receptors, thereby initiating downstream intracellular signalling cascades [77,78]. Hyaluronic acid, a naturally occurring high-molecular-weight glycosaminoglycan biopolymer, operates as a viscoelastic biomaterial scaffold and, simultaneously, as a ligand molecule for CD44 and as a receptor for hyaluronate-mediated motility (RHAMM) cellular surface receptors [79].

### 8.2. Individual Component Mechanisms and Integrated Therapeutic Synergy

Polynucleotide components orchestrate adenosine A_2_A receptor-mediated activation pathways, subsequently initiating intracellular signalling cascades that promote enhanced fibroblast proliferation, stimulate neovascular formation, and establish anti-inflammatory microenvironmental conditions via elevated interleukin-10 synthesis coupled with suppression of tumour necrosis factor-alpha expression [77]. Hyaluronic acid simultaneously augments cellular directional migration capacity, facilitates sequestration and retention of soluble growth factor mediators within defect anatomical sites, and maintains elevated aqueous phase composition within healing compartments [79]. Upon strategic combination within the Regenfast^®^ formulation, complementary synergistic mechanisms emerge: the hyaluronic acid matrix provides a biocompatible physical substrate, stabilising polynucleotide molecular bioavailability and therapeutic bioactivity, while incorporated polynucleotides reciprocally amplify hyaluronic acid-mediated fibroblast recruitment mechanisms and enhance angiogenic signalling pathways. Collectively, they establish a biphasic therapeutic microenvironment that simultaneously optimises soft-tissue organisational development and initiates early-phase osseous mineralisation processes [77].

### 8.3. Clinical Evidence and Therapeutic Applications in Peri-Implant Disease

Regenfast^®^ applications in peri-implant disease management remain inadequately characterised. Preclinical investigations demonstrate substantive periodontal tissue regenerative capacity when polynucleotides are administered for intrabony osseous defect management, with rabbit model studies documenting 60–70% improvement in volumetric bone fill relative to control sites [77]. Isolated hyaluronic acid administration demonstrates restricted therapeutic magnitude, primarily facilitating early-phase inflammatory resolution, particularly in diabetic patient populations [80]. The Regenfast^®^ combination formulation generated clinically significant improvements in deep intrabony osseous defects, achieving probing depth reductions of 3–4 mm and radiographic bone fill of 45–55% over six months [77]. Peri-implant applications remain limited. A rabbit maxillary sinus elevation study employing Bio-Oss^®^ supplemented with Regenfast^®^ demonstrated enhanced mineralisation kinetics at eight weeks relative to xenograft alone [77]. Prospective randomised clinical protocols evaluating Regenfast^®^ in peri-implant mucosal inflammation, peri-implantitis, or soft-tissue augmentation remain absent from peer-reviewed literature.

### 8.4. Cellular and Molecular Mechanisms of Therapeutic Activity

Polynucleotide components activate adenosine A_2_A receptors, while hyaluronic acid engages CD44 and RHAMM receptor populations [77,79]. These distinct receptor pathways enable multiplicative therapeutic amplification via independent intracellular signalling mechanisms [77]. The consolidated Regenfast^®^ formulation demonstrates synergistic soft-tissue regenerative benefits exceeding isolated component administration [76]. Emerging clinical data suggest amplified fibroblast recruitment, enhanced neovascularisation, and improved tissue matrix architecture compared with monotherapy [76]. Preclinical and clinical evidence indicates Regenfast^®^ promotes accelerated early-phase bone mineralisation within guided bone regeneration contexts, potentially facilitating improved osteogenic cell scaffold colonisation [76,77].

### 8.5. Substantive Evidence Limitations and Critical Knowledge Deficiencies

Long-term osseous regenerative capacity of Regenfast^®^ formulations remains scientifically unestablished [76,77]. Optimal timing parameters, dosimetric specifications, and patient selection criteria remain undefined. Rigorous comparative therapeutic effectiveness cannot be established due to absence of prospective randomised controlled protocols [4]. Polynucleotide biopolymer degradation kinetics in vivo remain inadequately characterised, limiting mechanistic comprehension of optimal dosimetric magnitudes and administration intervals [77].

### 8.6. Consolidated Therapeutic Understanding and Three Core Principles

Principle 1. Rational Synergistic Component Integration: Regenfast^®^ represents a scientifically justified combination strategy integrating immunomodulatory active polynucleotide components with structural biomaterial hyaluronic acid constituents, theoretically conferring therapeutic advantages relative to single-agent intervention approaches via complementary receptor activation mechanisms affecting independent signalling pathways [76,77].

Principle 2. Preferential Soft-Tissue Regenerative Profile: Accumulating clinical observational evidence indicates that Regenfast^®^ demonstrates preferential efficacy within soft-tissue healing developmental phases [77], while demonstrating measurable though not predominant osteogenic regenerative potential [76].

Principle 3. Investigational Status in Peri-Implant Disease Contexts: Regenfast^®^ maintains investigational therapeutic status for peri-implant disease management applications [4]. No dedicated prospective clinical investigation protocols evaluating its therapeutic effectiveness in peri-implant mucosal inflammation or advanced peri-implantitis disease are accessible within the scientific literature. Current clinical implementation patterns represent preliminary exploratory practice rather than evidence-based therapeutic decision making.

Regenfast^®^ represents a clinically accessible regenerative therapeutic option for general dental practitioners managing moderate-severity periodontal osseous defects and peri-implant soft-tissue anatomical complications. However, responsible clinical implementation necessitates comprehensive acknowledgment of existing evidence limitations, i.e., demonstrated therapeutic efficacy is limited to soft-tissue healing developmental phases and early-stage bone regenerative windows, with peri-implantitis disease management applications remaining empirical and speculative.

## 9. Direct Contradictions and Comparative Considerations 

### Critical Paradoxes Between Materials (Table 1)

The Growth Factor Paradox: Both FGF-2 and rhPDGF-BB promote bone regeneration (50–60% bone fill) yet operate through fundamentally distinct mechanisms [58,70]. FGF-2 inhibits osteogenic differentiation while promoting ligamentogenic responses, creating therapeutic tension [61]. Conversely, rhPDGF-BB simultaneously promotes osteogenic differentiation and soft-tissue proliferation [70,73]. This biological distinction suggests fundamentally different clinical profiles despite comparable bone fill outcomes [58,70].

The Scaffold Contradiction: GEM 21S^®^ employs β-TCP as an rhPDGF-BB delivery vehicle; however, β-TCP monotherapy paradoxically inhibits bone healing [72]. This apparent contradiction, employing an inhibitory scaffold to deliver osteogenic growth factor, reflects complex delivery kinetics and ion concentration effects requiring further mechanistic clarification [72].

The Multimodal Dependency Contradiction: EMD demonstrates robust efficacy when incorporated into multimodal protocols combining multiple biologically active agents, growth factors, and scaffolds [3]. Nevertheless, the same multimodal approach prevents the determination of EMD’s specific contribution, rendering true comparative effectiveness assessment impossible [4]. The apparent success may reflect cumulative effects of combined components rather than EMD-specific efficacy [4].

The Soft-Tissue–Bone Dissociation: Multiple materials (EMD, FGF-2, and rhPDGF-BB) enhance radiographic bone outcomes without proportional improvements in clinical parameters reflecting soft-tissue health (probing depth and bleeding on probing) [5,44,58]. This consistent dissociation suggests current regenerative approaches preferentially target osseous defects while leaving some inflammatory disease components unresolved [4].

**Table 1:** Biologically Active Materials on Peri-implant Diseases.

Material	Biological Source	Key Mechanism	Key Studies	Periodontal Evidence	Peri-Implant Evidence	Strongest Findings	Critical Limitations
Emdogain (EMD)	Porcine enamel matrix proteins (amelogenins)	Osteoblast proliferation; fibroblast stimulation; antimicrobial activity; and OPG/RANKL regulation	Froum et al. (2015) [3]; Isehed et al. (2016) [42]; Donos et al. (2023) [4]	Robust in intrabony defects; established clinical efficacy	Efficacy only within multimodal protocols; monotherapy ineffective	Multimodal approach: 5.1–5.4 mm PD reduction; 3.0–3.75 mm bone gain sustained for 3–7.5 years (Froum et al., 2015) [3]	Monotherapy fails to improve clinical parameters; no disease modification; requires multiple adjunctive agents; and lacks independent efficacy validation
Platelet-Rich Fibrin (PRF)	Autologous blood concentrate	Three-dimensional fibrin scaffold; progressive growth factor release (PDGF, VEGF, FGF, and TGF-β); and immunomodulation	Preclinical studies; periodontal regeneration literature	Demonstrated efficacy in periodontal regeneration; angiogenic effects	NO dedicated clinical trials for peri-implant mucositis or peri-implantitis	Autologous nature; progressive growth factor release; and immunomodulatory properties	Absence of peri-implant clinical validation; extrapolation from periodontal contexts; inconsistent outcomes in natural tooth applications; and undefined optimal protocols
Fibroblast Growth Factor-2 (FGF-2/bFGF)	Biotechnologically synthesised; approved as Trafermin in Japan (2016)	Fibroblast proliferation; angiogenesis; mesenchymal cell stimulation; and paradoxical osteogenic inhibition	Kitamura et al. (2016) [57]; Hyun et al. (2017) [63]; and Li et al. (2020) [61]	Phase III trials: 50.58% bone fill vs. 21.58% placebo; non-inferior to EMD	NO clinical trials for peri-implant disease	50.58% bone fill at 36 weeks; 1.927 mm linear alveolar bone growth; and modest CAL gains (~2 mm)	Inhibits osteogenic differentiation (paradox); dose-dependent effects; ligamentogenic bias; requires adjunctive SCOS for osteogenic restoration; and no peri-implant validation
rhPDGF-BB/GEM 21S	Biotechnologically synthesised PDGF-BB combined with β-TCP scaffold	Osteoblast mitogenic effects; macrophage-derived growth factor stimulation; direct chemotaxis; and simultaneous osteogenic and soft-tissue proliferation	Lynch et al. (1989) [66]; Nevins et al. (2005) [69]; Howell et al. (1997) [72]; and Choo et al. (2013) [73]	Multicentre RCTs: superior bone fill and CAL gains vs. placebo; ~2 mm CAL gain; and 43.2% defect fill	Pilot sheep study: superior bone regeneration in circumferential peri-implant defects; RCT on soft-tissue dehiscence (VCMX + rhPDGF-BB)	Comprehensive dual-regeneration (osteogenic + soft tissue); 2.08 mm new bone height; 43.2% osseous defect fill (Howell et al., 1997) [72]; and superior outcomes vs. β-TCP monotherapy	β-TCP paradoxically inhibits bone healing; discordance between in vitro/in vivo release kinetics; NO dedicated clinical trials for peri-implantitis; modest CAL gains despite superior bone fill; and expensive material
Regenfast (PN + HA)	Polynucleotides (fish sperm DNA) + hyaluronic acid	Dual-receptor activation (A_2_A adenosine; CD44/RHAMM); fibroblast proliferation; angiogenesis; anti-inflammatory response (IL-10 ↑; TNF-α ↓); and early mineralisation enhancement	Maniwa et al. (2024) [75]; retrospective periodontal series; and rabbit sinus lift model	Retrospective periodontal series: 3–4 mm PD reduction; 45–55% bone fill over 6 months; rabbit preclinical: 60–70% bone fill improvement	NO clinical trials for peri-implant mucositis or peri-implantitis; rabbit sinus lift: enhanced mineralisation at 8 weeks	Synergistic soft-tissue healing; PN promotes angiogenesis/fibroblast activity; HA provides viscoelastic scaffold; and dual-pathway activation	Long-term bone-regenerative capacity unproven; undefined dosing/timing; no RCTs; evidence confined to soft-tissue phases; PN degradation kinetics unclear; and speculative peri-implant application

## 10. Current Evidence Summary and Patient-Centred Outcomes

### 10.1. Biological and Surgical Evidence Synthesis

The investigation of biologically active materials in peri-implant disease management reveals a dichotomous evidence landscape: robust periodontal efficacy contrasted against markedly attenuated peri-implant validation [4]. Growth factors (rhPDGF-BB and FGF-2) demonstrate dose-dependent cellular proliferation and directional differentiation within periodontal contexts [70,81]; however, mechanistic translation to osseointegrated implant disease contexts remain fundamentally unvalidated, reflecting putative biological incompatibility rather than methodological investigation gaps [2]. Protein-derived materials (EMD) exhibit established periodontal regenerative capacity through enamel matrix protein-mediated cementoblast and periodontal ligament cell recruitment [3,5,44]; paradoxically, clinical peri-implant applications demonstrate marginal or absent efficacy, suggesting mechanistic inapplicability in non-periodontal-ligament-bearing tissues [4]. Formulations including Regenfast^®^ and PRF possess theoretical synergistic mechanisms—autologous immunocompatibility, temporally sustained growth factor liberation, leukocytic immunomodulation—yet clinical peri-implant validation remains conspicuously absent, representing evidence domains characterized by preliminary findings rather than robust prospective confirmation [4,23,77].

The persistent, quantifiable disparity between validated periodontal outcomes and negligible peri-implant therapeutic data reflects fundamental biological distinctions between these disease entities rather than insufficient clinical investigation intensity [2]. Peri-implant and periodontal pathologies exhibit mechanistically divergent regenerative requirements [2]. Natural dentition possesses inherent periodontal ligament architecture enabling three-dimensional tissue regeneration encompassing functionally integrated cementum, periodontal ligament, and alveolar bone compartments [82]. Osseointegrated implants fundamentally lack periodontal ligament structures, rendering therapeutic approaches predicated upon ligamentogenic differentiation and functional attachment restoration biologically inappropriate and mechanistically contradictory [2]. This constraint particularly constrains FGF-2 applicability, which demonstrates documented ligamentogenic bias through Runx2-modulated osteoblastic phenotype suppression and documented preference for fibroblastic lineage differentiation [61,67]. Similarly, protein-nucleotide-based biomaterial systems emphasizing inflammatory resolution mechanisms operate within biological contexts where established inflammatory infiltration resolution does not necessarily facilitate osseointegration restoration [61].

### 10.2. Patient-Centred Outcomes: Education, Literacy, and Behavioural Prevention

Modern peri-implant therapeutic paradigms necessarily integrate patient-centred educational and behavioural components, as surgical and biomaterial interventions demonstrate incomplete efficacy without sustained patient engagement [16,83]. The clinical literature increasingly acknowledges that material-centric approaches neglect the documented association between patient health literacy levels and long-term implant health trajectories [84]. Oral-health literacy—operationalised as the cognitive capacity to access, comprehend, critically appraise, and apply health-related information within disease prevention and management contexts—demonstrates quantifiable association with peri-implant disease incidence, progression kinetics, and treatment response variability [84]. Epidemiological evidence indicates that restricted oral-health literacy populations exhibit statistically significant elevations in peri-implant mucositis prevalence, accelerated progression toward advanced peri-implantitis phenotypes, and diminished clinical responses to both surgical intervention and antimicrobial therapy [84].

Structured patient education programmes incorporating implant-specific maintenance instruction, biofilm control methodologies calibrated to individual cognitive capacity, and symptom recognition frameworks demonstrate measurable efficacy in reducing peri-implant disease incidence and improving treatment compliance, particularly within populations with documented periodontitis history [16,84]. Behavioural prevention strategies—including motivational interviewing techniques, individualised risk stratification protocols, and temporally distributed reinforcement scheduling—demonstrate evidence-supported efficacy in sustaining long-term compliance with domiciliary oral hygiene protocols and professional maintenance appointment adherence [16,84]. Smoking cessation counselling and metabolic disease optimisation—particularly glycaemic control optimisation in diabetic patient populations represent evidence-validated behavioural intervention domains substantially modulating peri-implant disease susceptibility through documented immunocompetence optimization and inflammatory response modulation [84].

### 10.3. Digital Dissemination and Health Communication: Critical Quality Assessment

Digital health technologies ostensibly offer scalable, geographically distributed patient education opportunities; however, critical quality assessment reveals substantial heterogeneity in content accuracy, clinical evidence alignment, and information reliability, a methodological reality frequently obscured within promotional discussions of digital health implementation [84]. Systematic assessment of YouTube-hosted peri-implantitis educational content revealed profound accuracy deficiencies: only 38% of evaluated videos contained clinically accurate information aligned with current evidence-based peri-implant disease management protocols, whilst 42% demonstrated substantially misleading, incomplete, or contradictory information, and 20% presented content of indeterminate clinical utility [84]. Critically, video accuracy demonstrated inverse correlation with viewer engagement metrics and cumulative viewership, indicating that high-visibility, frequently accessed educational resources disproportionately contained inaccurate or clinically inappropriate information, thereby potentially compromising patient understanding and generating counterproductive behavioural responses [84].

This evidence gap substantiates the imperative for clinician-curated digital content development and strategic patient guidance toward evidence-validated, clinician-endorsed information sources rather than unfiltered digital platform dissemination [84]. Mobile health (mHealth) applications delivering personalised oral hygiene instruction and appointment reminders demonstrate efficacy in improving patient compliance with professionally recommended maintenance protocols [85]. Telehealth consultation modalities facilitate remote clinical assessment, particularly benefiting geographically disadvantaged or mobility-restricted populations; however, digital clinical assessment reliability remains incompletely characterised for peri-implant disease detection [86]. Automated patient portals facilitating secure clinical-patient communication enhance patient autonomy and information accessibility; however, portal implementation efficacy depends critically upon integration with established clinical protocols and clinician accessibility [87]. Future digital health implementation mandates incorporation of content verification mechanisms, clinician endorsement systems, and evidence-based integration pathways to ensure information dissemination integrity and clinical alignment [84].

### 10.4. Consolidated Understanding: Integrating Biological, Surgical, and Patient-Centred Dimensions

Optimal peri-implant disease management integrates materialised biological and surgical evidence with patient-centred education and rigorously vetted digital health implementation. Evidence-based clinical protocols should systematically incorporate:

(1) Comprehensive patient education addressing implant-specific maintenance requirements and biofilm control techniques, delivered through multimodal formats calibrated to documented individual health literacy levels [16,84];
(2) Behavioural prevention programmes implementing risk factor modification counselling, motivational interviewing, and temporally distributed compliance reinforcement [16,84];
(3) Critical evaluation and curation of digital resources, actively directing patients toward clinician-endorsed, evidence-validated information sources whilst providing explicit cautionary guidance regarding unreliable or inaccurate online content [84];
(4) Integrated digital health deployment utilizing mHealth applications and patient portals incorporating content verification mechanisms, clinician integration, and evidence-based protocol alignment [85,86,87].

Meta-analytical evidence indicates that comprehensive programmes systematically integrating patient education, behavioural interventions, and verified digital health technologies demonstrate superior long-term peri-implant health outcomes compared with conventional surgical treatment approaches conducted without parallel patient engagement optimization [16,84].

## 11. Conclusions

The therapeutic landscape of peri-implant disease management stands at a critical inflection point integrating biological, surgical, and patient-centred dimensions. While biologically active materials demonstrate compelling regenerative capacity within periodontal contexts [1,2,3], their translation to osseointegrated implant treatment represents not merely an evidence gap but rather a biological mismatch requiring fundamental reconsideration [9]. The persistent disparity between robust periodontal validation and negligible peri-implant efficacy data [4,5,6,7,8] reveals an uncomfortable scientific truth: what may work with natural teeth may not work with dental implants, and these materials have fundamentally different therapeutic indications [9,10]. This is not a matter of insufficient clinical investigation but rather an issue of mechanistic incompatibility: therapeutic agents optimised for periodontal tissue differentiation and cementum neodeposition [11] operate within a biological context where such structures do not exist post-osseointegration.

The current evidence suggests that contemporary biologically active material platforms, including recombinant growth factors [2,3], enamel matrix derivatives [4,5,6], and formulations such as Regenfast^®^ and PRF [1,7,8]—show promising mechanistic potential yet remain investigational for peri-implantitis management. While FGF-2’s osteogenic pathways and rhPDGF-BB’s soft-tissue augmentation mechanisms demonstrate biological activity, their clinical efficacy in established peri-implantitis contexts requires further validation. Multicentre randomised controlled trials stratifying outcomes across peri-implant mucosal inflammation, early inflammatory osseous disease, and advanced circumferential defects would substantially advance evidence-based application. Pending such investigations, practitioners are advised to integrate these materials within established surgical fundamentals—plaque removal, meticulous decontamination, strategic bone grafting, and optimised wound and soft-tissue management—while recognizing their current investigational status [12].

Comprehensive peri-implant disease management increasingly reflects evidence supporting integration of patient-centered educational and behavioural interventions alongside surgical approaches. Patient education addressing implant-specific maintenance, oral-health literacy, and behavioural prevention strategies demonstrates evidence of contributing to favourable long-term outcomes [83,84]. Emerging digital platforms—including mHealth applications, telehealth services, virtual reality education, and wearable intraoral sensors—offer scalable pathways for patient engagement and monitoring, potentially enhancing peri-implant disease prevention [85,86,87].

Biologically active materials may serve as complementary adjuncts rather than standalone interventions within comprehensive, evidence-informed surgical protocols. This approach encourages scientific objectivity regarding material efficacy, measured clinical communication, and commitment to patient-centred outcomes extending beyond surgical resolution to include sustained implant health maintenance [4,5,6]. Future research should prioritize randomised controlled trials evaluating peri-implantitis-specific applications, concurrent assessment of patient education and behavioural intervention efficacy, and digital health technology integration, thereby establishing robust evidence for comprehensive, patient-centred peri-implant disease management.

## Data Availability

The data supporting the findings of this study are available within this article, as detailed in the relevant tables presented in this manuscript. FM had full access to all the data in this study and takes complete responsibility for the integrity of the data and the accuracy of the data analysis.
